# Development and testing of compact electronic modules for detectors based on SiPM array

**DOI:** 10.12688/openreseurope.19256.1

**Published:** 2025-01-31

**Authors:** Ramil Akbarov, Sabuhi Nuruyev, Sergei Tyutyunnikov, Patrik Kucera, Michael Holik

**Affiliations:** 1 Innovation and Digital Development Agency, Nuclear Research Department, IDDA, Gobu str. 20th km of Baku-Shamakhi Highway, Baku, AZ01021, Azerbaijan; 2 Institute of Radiation Problems under Ministry of Science and Education, B.Vahabzade str. 9, Baku, AZ1143, Azerbaijan; 3 Joint Institute for Nuclear Researches, Joliot-Curie 6, Dubna, 141980, Russian Federation; 4 Khazar University, 41 Mahsati Str., Baku, AZ1096, Azerbaijan; 5 Faculty of Electrical Engineering, UWB in Pilsen, Univerzitni, 2795/26, Pilsen, 306 14, Czech Republic

**Keywords:** SiPM, MAPD-3NM, electronic modules, scintillation detector

## Abstract

**Background:**

Compact electronic modules are essential for modern detector systems utilizing Silicon Photomultiplier (SiPM) arrays due to their small size, low power consumption, and high precision. To address the growing demand for portable and efficient detection systems, the development of modules tailored for such applications has become a critical focus. This study introduces compact electronic modules designed for detectors based on MAPD (Microcell Avalanche Photodiode) arrays, aiming to improve reliability and versatility for industrial, medical, and scientific applications.

**Methods:**

The developed modules include two primary components:

DC-DC Voltage Converter: Based on the MAX1932ETC chip.

Converts a 5 V input to a stable output voltage adjustable between 30 and 90 V.

Supports a maximum current of 2.5 mA, ensuring reliable operation for SiPM arrays.

Signal Amplification Unit: Utilizes the LTC6268 chip.

Processes input signals ranging from 10 to 75 mV.

Offers a gain factor of 45, characterized by low noise and high precision.

The modules were integrated with a scintillation detector comprising a MAPD-3NM-II photodiode array and an LFs scintillator. Performance testing was conducted using a Cs-137 gamma source.

**Results:**

The energy resolution of the detector system was evaluated using the 662 keV gamma line from the Cs-137 source. The modules achieved an energy resolution of 10 ± 0.5%, demonstrating high reliability and efficiency. This performance confirms the modules’ capability to maintain stable operation and precise signal processing under real-world conditions.

**Conclusions:**

The developed compact electronic modules provide a cost-effective and efficient solution for detectors utilizing SiPM arrays. Their stable voltage conversion, low-noise signal amplification, and high energy resolution make them suitable for a wide range of applications in industrial, medical, and scientific fields. Future work may focus on optimizing the modules for broader voltage ranges and compatibility with different scintillator materials to expand their applicability.

## Plain Language Summary

This study describes the development of small, efficient electronic modules designed for detectors using advanced light sensors called Silicon Photomultipliers (SiPMs). These modules include two key parts:

A voltage converter that turns a 5V input into a stable, adjustable output between 30 and 90 volts, allowing the detector to operate reliably.

A signal amplifier that boosts weak signals from the detector, making them clearer and easier to measure.

The modules were tested with a special detector that uses an MAPD-3NM-II light sensor and a material called LFs, which glows when it detects radiation. Using a radioactive source (Cs-137), the detector measured the energy of gamma rays with high accuracy, achieving an energy resolution of 10%.

These results show that the modules are reliable and effective. They are compact, affordable, and suitable for use in fields like medical imaging, industrial safety, and scientific research.

## Introduction

Silicon photomultipliers (SiPMs) ^1- 3^ have emerged as a transformative technology in the field of photodetection, offering significant advantages over traditional vacuum photomultiplier tubes (PMTs). Their compact design, low operating voltage, fast response, and high sensitivity have made SiPMs an essential component in diverse applications, including particle physics, astrophysics, nuclear physics, medical imaging, and security systems ^4– 10^. SiPM-based detectors, particularly in scintillation applications, are gaining prominence in high-energy physics due to their ability to register particles across a broad intensity spectrum ^11^. Furthermore, their integration into positron emission tomography (PET) scanners as matrix assemblies has demonstrated the potential to enhance photon detection efficiency in medical diagnostics ^12^.

Matrix assemblies of avalanche photodiodes, such as the MAPD (Micro-pixel Avalanche Photodiode), are especially advantageous in experiments requiring large active areas for effective photon registration. However, the implementation of SiPM matrices necessitates the development of specialized electronic modules to ensure optimal performance ^13– 15^. These modules typically include power supply systems capable of providing stable, adjustable high voltages and signal amplification units designed for low-noise operation with high gain factors. Despite the growing interest in SiPM-based systems, the availability of compact, cost-effective, and high-performance electronic modules for such applications remains limited.

This study addresses this gap by presenting the development and testing of compact electronic modules specifically designed for detectors utilizing MAPD arrays. The developed modules include a DC-DC voltage converter capable of delivering a stable output voltage within the range of 30–90 V and a signal amplification unit that amplifies input signals with a gain factor of 45. To evaluate their performance, the modules were integrated into a scintillation detector comprising a MAPD-3NM-II photodiode matrix ^16^ and a LSO scintillator. Testing with a Cs-137 gamma source demonstrated the modules’ effectiveness and reliability, showcasing their potential for use in industrial, medical, and scientific applications.

## Methods

### DC-DC converter

**Figure 1.**
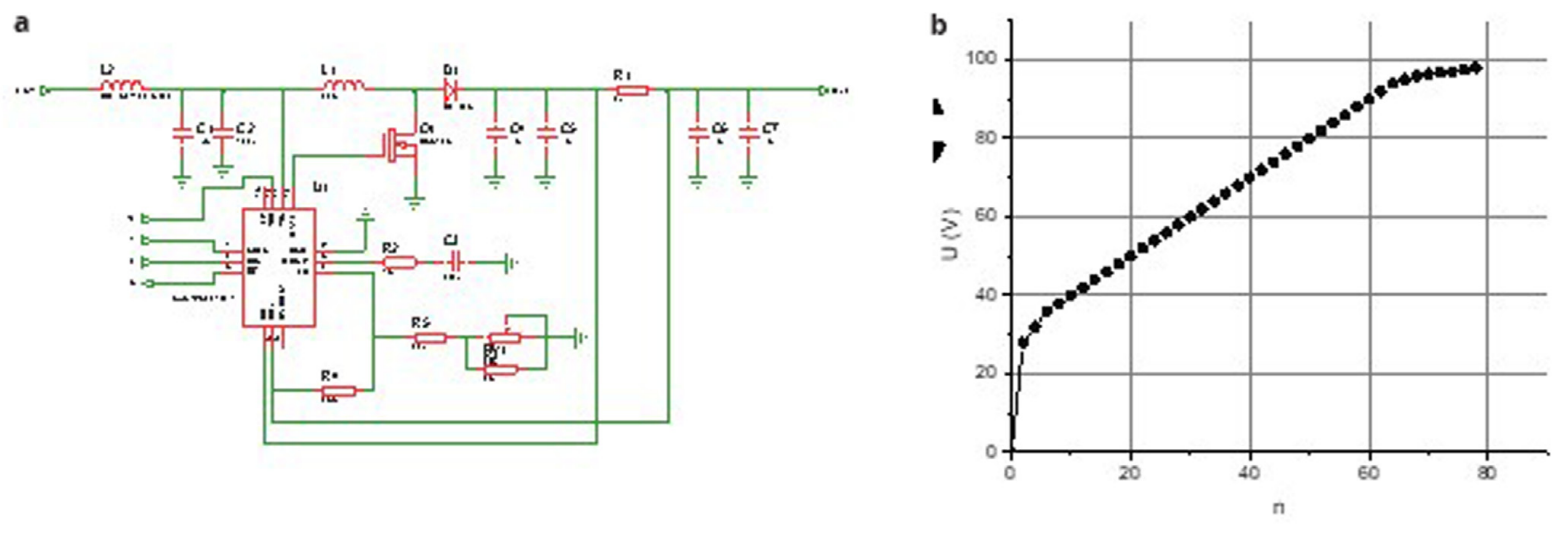
Circuit diagram of the DC-DC converter ( **a**) and the dependence of the output voltage on the resistance of the regulator ( **b**).

The matrix utilized in this study consisted of 16 individual MAPD elements arranged in a 4×4 configuration, resulting in a total matrix size of 14.8×14.8 mm ^2^. Each MAPD element operates at a nominal voltage of 54 V, necessitating a power supply capable of providing stable and adjustable high-voltage output. To meet these requirements, a DC-DC converter circuit was designed and assembled using the MAX1932ETC chip, known for its high efficiency and reliability in voltage conversion applications ( Figure 1a). The converter was configured to accept a standard 5 V input, a convenient and widely available supply voltage, and to produce a regulated output voltage within the range of 30 to 90 V. The circuit is characterized by its simplicity and exceptional stability, offering reliable operation under varying conditions.

One of the key features of this circuit is its capability for precise voltage adjustment using a potentiometer, allowing for fine-tuning to match specific operational requirements. Additionally, the design includes the option to connect a thermistor to modify the output voltage dynamically, an important advantage given the sensitivity of SiPMs to temperature variations. To evaluate the performance of the converter, test measurements of the output voltage as a function of the regulator resistance were conducted, and the results are presented in Figure 1b. The circuit demonstrated power consumption of less than 2 mA, indicating its efficiency. Furthermore, a linear relationship between the output voltage and the normalized resistance was observed across the adjustable range of 30 to 90 V, with a maximum current limit of 2.5 mA. These characteristics highlight the converter’s excellent performance, making it well-suited for powering SiPM arrays in high-precision applications.

### Signal amplifier

To enhance the output signal of the MAPD matrix, a signal amplification circuit was designed and assembled using the LTC6268 chip. This amplifier is specifically chosen for its combination of low power consumption and minimal noise generation, making it highly suitable for applications demanding precision and sensitivity. These include photodiode signal processing, measurement systems, and detailed signal analysis. Additionally, its compact design ensures its seamless integration into systems requiring single-channel configurations, further enhancing its versatility for various high-performance applications.

**Figure 2.**
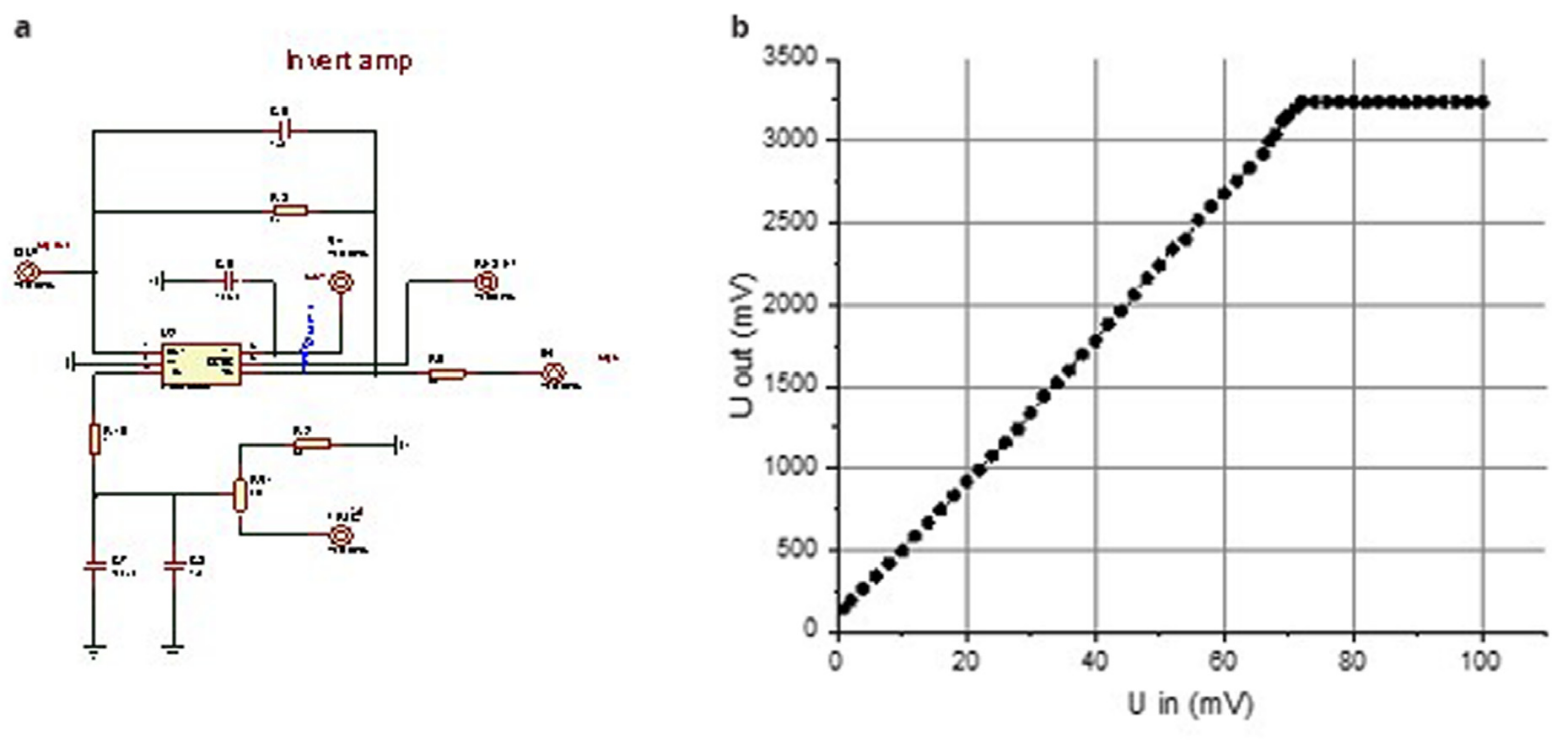
Circuit diagram of the inverting preamplifier ( **a**) and the dependence of the output amplitude on the input signal amplitude ( **b**).

The circuit operates by connecting the photodiode to the inverting input of the operational amplifier through a low-resistance resistor, designated as R9 ( Figure 2a). The photodiode's output current is converted into a corresponding voltage signal by the feedback resistor R8, which plays a pivotal role in defining the circuit's gain. The gain of the amplifier is governed by the ratio of the resistances R8 and R9, with these values carefully selected based on the anticipated signal levels from the photodiode. To further optimize performance, a feedback capacitor, labeled C5, is integrated into the circuit to suppress unwanted noise and mitigate high-frequency oscillations that could compromise signal integrity.

The circuit is powered by a voltage supply ranging from 2.7 V to 5 V, making it compatible with both battery-based systems and constant voltage sources. This flexibility allows it to be deployed in a variety of operational scenarios. The output voltage V _out_ and the amplifier gain K are determined by well-established mathematical expressions below, linking these parameters to the current generated by the photodiode and the resistor values. Overall, this amplifier circuit provides a robust, efficient, and precise solution for enhancing the signals generated by MAPD matrices in demanding detection and measurement environments.


$$V_{out}=I_{ph}\times R8,\;K=\frac{R8}{R9},$$

where I _ph_ is the current generated by the photodiode.

The gain can be determined from the dependence of the output amplitude on the input signal amplitude ( Figure 2b). In this case, the gain K is 45, with a bandwidth of approximately 100 MHz. The linear range of input amplitudes for amplification was from 10 mV to 75 mV. This range allows the use of photodiode matrices with output amplitudes in the tens of millivolts.

### Testing of electronic modules

**Figure 3.**
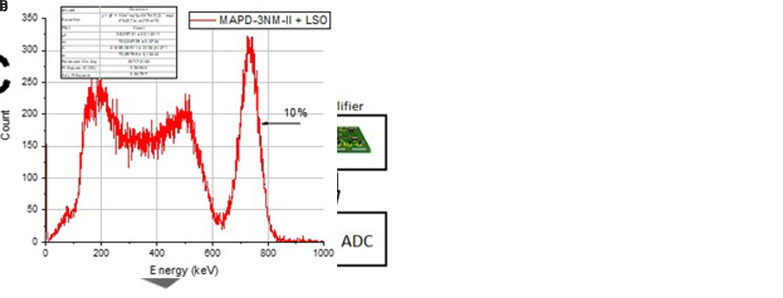
Test experimental setup ( **a**); Cs-137 spectrum obtained by the test detector based on the MAPD array+LSO ( **b**).

The electronic modules were tested using a scintillation detector based on a MAPD-3NM-II avalanche photodiode array and a LSO scintillator ^17^. The crystal dimensions were 15×15×15 mm ^3^. To minimize light losses, the scintillator was covered with Teflon tape. One side of the scintillator was left open for connection with the SiPM matrix ( Figure 3a). To ensure better optical contact between the MAPD matrix and the scintillator, a special optical gel with a high transparency coefficient was used.

To test the capability of registering ionizing radiation, a calibration point source of cesium Cs-137 was used, positioned above the scintillator. The DC-DC converter converted 5V into a stable 54V, which powered the matrix. The signal from the matrix was amplified by the assembled amplifier with a gain of 45. The data were recorded and analyzed using the CAEN DT5720 analog-to-digital converter (ADC) with a sampling rate of 250 MHz. Measurements were conducted at room temperature in a light-isolated chamber.

The acquired data are presented in the spectrum shown in Figure 3b. Data analysis revealed an energy resolution of 10 ± 0.5% for the 662 keV gamma emission of Cs-137. This level of precision indicates a low noise level in the developed electronic modules. Despite their simplicity, the electronic modules demonstrate high performance.

## Conclusion

Highly efficient electronic modules were designed and developed to support the operation of avalanche photodiode (MAPD) matrices, ensuring reliable performance in detection systems. The DC-DC converter module is capable of delivering a stable and adjustable output voltage within the linear range of 30 to 90 V, with a high precision of up to 10 mV and a current capacity of up to 2.5 mA. This capability provides the necessary flexibility to meet the voltage requirements of various MAPD configurations. Additionally, the amplification module is designed to enhance input signals ranging from 10 to 75 mV, achieving a fixed gain of 45. These characteristics make the modules particularly well-suited for detectors utilizing MAPD arrays, enabling accurate and sensitive signal processing.

The modules were rigorously tested within a scintillation detection system, which comprised a MAPD matrix and a LSO scintillator. A calibration experiment was conducted using Cs-137 as the gamma source, emitting gamma quanta with an energy of 662 keV. The testing demonstrated an impressive energy resolution of 10 ± 0.5%, underscoring the low-noise performance and precision of the developed modules. These results validate the modules' effectiveness in amplifying and stabilizing signals in complex detection scenarios.

In conclusion, the developed electronic modules represent a compact, efficient, and cost-effective solution for powering and processing signals from silicon avalanche photodiode matrices. Their demonstrated performance makes them highly suitable for a wide range of applications in industrial, medical, and scientific fields, including radiation detection, medical imaging, and particle physics experiments. The modules’ compact design and robust functionality further enhance their potential for integration into advanced detection systems, meeting the demands of high-precision and high-sensitivity applications.

## Data Availability

Open Science Framework: Development and Testing of Compact Electronic Modules for Detectors Based on SiPM array. https://doi.org/10.17605/OSF.IO/FZ6J9 ^18^ This project contains the following data: Amplifier data.xlsx (the dependence of the output amplitude on the input signal amplitude) Power supply data.xlsx (the dependence of the output voltage on the resistance of the regulator) Spectr Cs-137 -MAPD-3NM2-LSO.xlsx (Cs-137 spectrum obtained by the test detector based on the MAPD array+LSO) Open Science Framework: Development and Testing of Compact Electronic Modules for Detectors Based on SiPM array. https://doi.org/10.17605/OSF.IO/FZ6J9 ^18^ This project contains the following data: Amplifier circuit.eps (circuit diagram of the inverting preamplifier) Amplifier data.eps (the dependence of the output amplitude on the input signal amplitude) Experiment scheme.tif (test experimental setup) Power supply circuit.eps (circuit diagram of the DC-DC converter) Power supply data.eps (the dependence of the output voltage on the resistance of the regulator) Spectr img.eps (Cs-137 spectrum obtained by the test detector based on the MAPD array+LSO) Data are available under the terms of the Creative Commons Zero "No rights reserved" data waiver (CC0 1.0 Public domain dedication) ( http://creativecommons.org/publicdomain/zero/1.0/)
