# Supplementary material for: Development and testing of compact electronic modules for detectors based on SiPM array
Source: Open Res Eur. 2025 Jan 31;5:33. [Version 1] doi: 10.12688/openreseurope.19256.1 (PMC12495388; doi:10.12688/openreseurope.19256.1)
Supplement: Peer review history

# Reviewer report: Reviewer response for version 1

**DOI:** 10.21956/openreseurope.20843.r61233

**Published:** 2025-10-03

**Authors:** Azer Sadigov

**Affiliations:** 1 Department of Nuclear Research of IDDA, Baku, Azerbaijan

The authors present a comprehensive study on the development and testing of compact electronic modules for SiPM array–based detectors.

 The design concept is clearly described, with a strong focus on low-noise front-end electronics, optimized signal shaping, and fast readout integration.

 These features are essential to fully exploit the intrinsic advantages of SiPM technology, such as high photon detection efficiency and fast timing response. The authors also address critical challenges associated with SiPM operation, including suppression of dark counts, ensuring linearity of response across a wide dynamic range, and minimizing inter-pixel cross-talk.

 The testing methodology—based on calibration with standard radiation sources and detailed evaluation of gain stability and timing resolution—demonstrates both scientific rigor and practical orientation.

 One of the major strengths of the work lies in the compactness of the developed modules. This design feature opens clear perspectives for their integration into portable and field-deployable detection systems, which is of great importance for medical imaging, homeland security, and environmental monitoring. The modular structure further enhances flexibility, allowing the system to be adapted to different array sizes and geometries. Such scalability ensures applicability in a wide variety of experimental and applied contexts. The demonstrated stable operation under different environmental conditions highlights the robustness of the proposed solution and strengthens its potential for real-world deployment.

 At the same time, the review notes certain limitations. **The figures presented in the work are of relatively low resolution**, which reduces the clarity of the technical details and may hinder a full assessment of the results. Improving figure quality would significantly enhance the overall readability and impact of the study.

Is the study design appropriate and does the work have academic merit?

Yes

Is the work clearly and accurately presented and does it cite the current literature?

Yes

If applicable, is the statistical analysis and its interpretation appropriate?

Partly

Are all the source data underlying the results available to ensure full reproducibility?

Yes

Are the conclusions drawn adequately supported by the results?

Partly

Are sufficient details of methods and analysis provided to allow replication by others?

Partly

Reviewer Expertise:

Semiconductor physics, Radiation detectors, Nuclear research

I confirm that I have read this submission and believe that I have an appropriate level of expertise to confirm that it is of an acceptable scientific standard.

# Reviewer report: Reviewer response for version 1

**DOI:** 10.21956/openreseurope.20843.r50651

**Published:** 2025-07-18

**Authors:** Sonali Bhatnagar

**Affiliations:** 1 Dayalbagh Educational Institute, Agra, India

The paper is based on the electronics experimental design of Silicon photo multiplier arrays for scintillation detectors. This is an important area of research that shall minimise the size and increase the efficiency of the arrays. This shall be a boost to multi-messenger astrophysics, which is the present-day science of the Universe. These new experimental studies shall support the astrophysics, medical science, and space physics research where the size of the instrument is an important parameter.

 The work is clear and accurate for the SiPMTs data given. I have seen the data sheets of the amplifier, power supply, and spectrum of Cs-137.

 The work has academic merit as required in astroparticle physics.

 Few points need to be clarified:

 1.In Fig. 1, the second graph depicts the output voltage vs resistance of the regulator. The resistance is not given with units as it is normalised, so mention this in caption. 

 2. The Fig.1 a can be  shown with a large font size to make it  clear.

 3. The Figure 2 a and 2b need to be more clear with large font size in labels of the circuit and axes labels.

 4. In line "The linear range of input amplitudes for amplification was from 10 mV to 75 mV. , the  range looks to be 10 mV to 70 mV, not 75mV.

 5. a special optical gel with a high transparency coefficient was used, mention this gel and value of coefficient.

 6. Fig 3b inset details are not clear in the spectrum.

 The energy spectrum is supported by data, and energy resolution has been clearly shown. The source data has been made available, and the conclusions are appropriate.

Is the study design appropriate and does the work have academic merit?

Yes

Is the work clearly and accurately presented and does it cite the current literature?

Yes

If applicable, is the statistical analysis and its interpretation appropriate?

Yes

Are all the source data underlying the results available to ensure full reproducibility?

Yes

Are the conclusions drawn adequately supported by the results?

Yes

Are sufficient details of methods and analysis provided to allow replication by others?

Yes

Reviewer Expertise:

Detector Physics, astroparticle physics

I confirm that I have read this submission and believe that I have an appropriate level of expertise to confirm that it is of an acceptable scientific standard.
